# Higher Plasma Sphingosine-1-Phosphate Levels in Type 2 Diabetic Patients Have a Non-Linear Relationship with the Disease Prognostic Indices and Microvascular Complications: A Cross-Sectional Saudi Study

**DOI:** 10.3390/jcm15093233

**Published:** 2026-04-23

**Authors:** Basil M. Alomair

**Affiliations:** Department of Internal Medicine, College of Medicine, Jouf University, Sakaka 72388, Saudi Arabia; bmalomair@ju.edu.sa; Tel.: +966-535115185

**Keywords:** sphingosine-1-phosphate, type 2 diabetes, complications, biomarker potential, non-linear relationships, metabolic risk

## Abstract

**Background/Objectives:** Sphingosine-1-phosphate (S1P) is implicated in glycemic control. However, its circulating levels and clinical significance in type 2 diabetes mellitus (T2DM) remain controversial. We assessed plasma S1P levels in T2DM patients, its associations with metabolic parameters and complications, and explored its biomarker potential and non-linear (U-/J-shaped) relationships. **Methods:** This cross-sectional study enrolled 140 patients with T2DM and 63 matching healthy controls. Plasma S1P was measured by competitive ELISA. Statistical analyses included comparisons, correlation, ROC analysis, multivariable logistic regression, and quadratic/spline regression for U-shaped relationships. **Results:** Plasma S1P was significantly elevated in T2DM patients [1256.7 (149.4–1510.0) ng/mL] compared to controls [1075.1 (202.0–1510.0) ng/mL; *p* < 0.001]. S1P correlated positively with age, disease duration, HbA1c, insulin resistance, TyG index, triglycerides, systolic blood pressure, and negatively with HDL-C. Patients with complications had higher S1P than those without (*p* = 0.001), with progressive increases from retinopathy to nephropathy to mixed complications. Insulin-treated patients exhibited the highest S1P levels (*p* < 0.001). ROC analysis showed moderate diagnostic accuracy (AUC = 0.724). S1P is an independent associated factor with complications (OR = 1.18 per 100 ng/mL, *p* = 0.003). Non-linear analysis revealed a U-shaped relationship with HDL-C (optimal S1P: 1100–1350 ng/mL) and a J-shaped relationship with complication risk (threshold ~1250 ng/mL). **Conclusions:** Plasma S1P is elevated in T2DM and correlates with disease severity, glycemic control, insulin resistance, and complications. S1P demonstrates moderate biomarker potential and exhibits non-linear U-/J-shaped relationships with metabolic parameters, suggesting an optimal therapeutic window of 1100–1280 ng/mL. These findings support S1P as a marker of cumulative disease burden and a potential therapeutic target.

## 1. Introduction

Type 2 diabetes mellitus (T2DM) is one of the most prevalent metabolic disorders worldwide and is driven by chronic hyperglycemia, insulin resistance, and progressive β-cell dysfunction. Despite advances in understanding its pathophysiology and expanding treatment options, these efforts remain insufficient to curb the national and global increase in its prevalence and devastating complications [1,2]. The dyslipidemic state characteristic of T2DM, driven by free fatty acid overload, not only induces pancreatic β-cell lipotoxicity and insulin resistance but also accelerates sphingolipid synthesis. Among these lipid mediators, the homeostasis of key sphingolipid intermediates, particularly ceramides and sphingosine-1-phosphate (S1P), has emerged as critically important, functioning both as receptor ligands and intracellular secondary messengers with profound metabolic, immunological, and cardiovascular implications [3,4].

S1P is a bioactive sphingolipid metabolite synthesized from sphingosine via two distinct sphingosine kinase (SphK) isoforms: SphK1 and SphK2. These isoforms differ markedly in their intracellular localization, regulation, signaling mechanisms, tissue expression, and biological functions [3,5]. S1P is irreversibly degraded by S1P lyase or reversibly dephosphorylated by S1P phosphatases [6]. It exerts its pleiotropic autocrine, paracrine, or endocrine effects primarily through five S1P G protein-coupled receptors (S1PR1-5), activating diverse downstream signaling cascades that influence inflammation, autophagy, insulin signaling, adiposity, and cell fate [7].

Circulating S1P is derived from erythrocytes, platelets, and endothelial cells through specific transporters (Mfsd2b and SPNS2) [8]. In plasma, approximately 70% of S1P is bound to apolipoprotein M (apoM) within a small fraction of high-density lipoprotein (HDL; mainly HDL3) particles, whereas the remaining 30% is associated with albumin [9]. This differential carrier distribution profoundly influences S1P’s biological activity: HDL/apoM-bound S1P exhibits a four-fold longer half-life and signals preferentially through S1PR1 and S1PR3, exerting vasoprotective and anti-atherogenic effects. In contrast, albumin-bound S1P preferentially activates S1PR2, which may promote pro-inflammatory and insulin-antagonistic responses [4,8]. Therefore, the regulation of glucose and lipid metabolism by the SphK/S1P axis, with both beneficial and detrimental effects, depends on the cellular context, receptor engagement, and disease stage. S1P levels are elevated in obese adipose tissue, where it functions as a pro-inflammatory mediator through S1PR2, promoting adipocyte hypertrophy, lipolysis, and macrophage recruitment [3,10]. Genetic or pharmacological SphK1 inhibition attenuates adipose inflammation and improves systemic insulin sensitivity [10]. Conversely, S1PR2 antagonism enhanced insulin sensitivity in obese mice [11].

In skeletal muscle, S1P enhances glucose uptake and insulin signaling, and exercise increases both interleukin (IL)-6 and S1P levels, where S1P stimulates IL-6 production via S1PR3 [12,13]. In the liver, SphK1 expression is elevated in steatosis, whereas hepatic SphK2 expression is reduced in T2DM patients [14,15]. The SphK/S1P axis critically regulates pancreatic β-cell function. S1P enhances insulin secretion, and SphK1 knockdown reduces insulin gene transcription and content [16]. Glucose stimulates SphK2-mediated S1P production, which correlates with increased insulin secretion [17]. However, intracellular S1P accumulation can also induce β-cell dysfunction and apoptosis, suggesting that extracellular S1P is protective, whereas intracellular S1P may be detrimental [4,18]. The adipokine, adiponectin, activates ceramidase, increases S1P production, and prevents β-cell and cardiomyocyte apoptosis [19]. Notably, SphK1 and SphK2 exhibit opposing roles in diabetes: global SphK1 deletion promotes β-cell loss and insulin resistance, whereas SphK2 deficiency protects against lipo-apoptosis and diabetes [20,21]. Circulating dihydro-S1P (dhS1P), a product preferentially generated by SphK2, is strongly associated with T2DM risk, implicating SphK2 activity in disease pathogenesis [22].

S1P plays a complex role in diabetic microvascular and macrovascular complications. HDL/apoM-bound S1P, acting via S1PR1, supports endothelial barrier function and suppresses pathological angiogenesis, whereas albumin-bound S1P may promote pro-atherogenic effects via S1PR2 [8]. Plasma S1P levels are reduced in atherosclerosis but elevated in T2DM, with conflicting reports regarding their association with cardiovascular disease [23,24]. Plasma S1P and apoM levels are independently and negatively associated with mortality in African American T2DM patients [25]. In diabetic nephropathy, urinary S1P is elevated and correlates with proteinuria, whereas serum apoM is inversely correlated with nephropathy stage, suggesting reno-protective roles [26,27]. S1PR4 antagonism protects against podocyte loss and fibrosis, whereas S1PR4 overexpression induces podocyte death [28]. In retinopathy, plasma S1P is inversely correlated with disease severity, and SphK inhibition protects against light-induced retinal degeneration [29,30]. Plasma HDL from T2DM patients has reduced S1P content and an impaired ability to stimulate endothelial nitric oxide synthase (eNOS) and to suppress inflammation, effects that are reversible by S1P enrichment [31,32]. In vitro glycation of HDL impairs S1P binding, although this has not been observed in diabetic humans despite reduced apoM levels [33].

The differential effects of S1P on insulin resistance may be explained by its carrier proteins. S1P-apoM/HDL preferentially activates S1PR1/S1PR3, supporting Akt-mediated insulin signaling, whereas S1P-albumin activates S1PR2, antagonizing insulin action. This effect is abolished by the S1PR2 antagonist JTE-013 [4,34]. Plasma apoM levels are inversely associated with insulin resistance and are lower in obesity, whereas elevated S1P characterizes diabetes, suggesting a shift toward albumin-bound S1P under pathophysiological conditions [35]. Fingolimod, a structural S1P analog, improves glucose homeostasis and insulin sensitivity in animal models, although its effects are complex owing to both agonistic and antagonistic properties [12,36]. Myriocin, which inhibits sphingolipid synthesis, improves insulin sensitivity and lipid profiles in diet-induced insulin resistance [37]. Emerging evidence suggests that the relationship between circulating S1P and clinical outcomes may be non-linear, which may reflect the existence of an optimal S1P level within a narrow therapeutic window [38,39,40,41].

We targeted a Saudi population with a largely sedentary lifestyle and energy-dense nutrition that is reflected in one of the highest prevalence of diabetes worldwide. Significant gaps remain in our understanding of the clinical significance of circulating S1P in T2DM as inferred from the aforementioned paradox [5]. Moreover, the relationship between S1P levels and key metabolic parameters, blood pressure, disease complications, and treatment remains incompletely characterized. The possibility of a U-shaped relationship between S1P and metabolic outcomes in T2DM has not been systematically examined. We planned to address these issues in a cross-sectional study.

## 2. Materials and Methods

### 2.1. Study Design and Setting

This cross-sectional study enrolled T2DM patients who were diagnosed and followed up at the Diabetes Outpatient Clinic of the Diabetes Center, King Abdulaziz Specialized Hospital, Sakaka, Al-Jouf, Saudi Arabia, in the period from 1 July to 30 December 2023. We evaluated the associations and patterns between variations in plasma S1P levels and disease duration, type of treatment, hypertension, glycemic control, lipid profile, insulin resistance, and microvascular complication profile in patients with T2DM at a single time point, and assessed S1P biomarker potential. The study was bioethically approved by the Permanent Research Ethics Committee of Jouf University, Sakaka, Saudi Arabia (Approval# 3-09-44 on 19 June 2023), enrolled informed and written-consented participants, and adhered to the tenets of the 2024-revised Declaration of Helsinki.

### 2.2. Study Participants and Sampling

Two distinct groups were concurrently enrolled to provide a comparative baseline for T2DM adult patients (≥18 years) with a clinically confirmed hospital-based diagnosis versus sociodemographically, sex-, age-, and BMI-matched healthy control adults with no history of diabetes or prediabetes. Controls were included to establish a reference range for physiological S1P levels in our population. Assessment of S1P’s biomarker discriminatory power, through receiver operating characteristic (ROC) curve analysis, also necessitates the presence of healthy controls. The sample size was calculated using Calculator.net (https://www.calculator.net/sample-size-calculator.html?type=1&cl=95&ci=5&pp=25&ps=400000&x=68&y=16, accessed on 28 February 2026) to be 203 participants, with a margin of error of 5%, a Sakaka adult population of 75,000, and a T2DM disease prevalence of 25%. Sample size suitability was confirmed by post hoc and power analyses based on our observed outcomes. With a recruited sample of 140 T2DM patients and 63 healthy controls, and an observed Area Under the Curve (AUC) of 0.724 for S1P, the study achieved >95% statistical power to detect a significant deviation from the null hypothesis (AUC = 0.50) at a 5% significance level (two-tailed α = 0.05). Furthermore, this sample size provided >95% power to detect the highly significant differences in continuous plasma S1P levels observed between the two cohorts. After signing the informed consent form, participants were enrolled sequentially until the target sample size was attained. Patients with chronic kidney and liver failure, autoimmune diseases (including T1DM), aggressive diabetes complications (diabetic foot and gangrene or current ketoacidosis), all types of cancer, pregnancy, immobility, and inflammatory effects unrelated to T2DM were excluded.

### 2.3. Investigations and Data Collection

The relevant de-identifying sociodemographic and anthropometric medical record data collected include: sex, age, routine laboratory investigations (fasting glucose, HbA1c, lipid profile [total, LDL- and HDL-cholesterol, and triglycerides], and total WBC count), micro- and macro-vascular complications (complication-free, microvascular [ophthalmopathy, nephropathy, or neuropathy], and macrovascular [cardiac and stroke]), disease duration, and, type of current treatment (naïve, diet control, metformin, hypoglycemic ± metformin, insulin ± hypoglycemic and/or metformin). The triglyceride–glucose insulin resistance index (TyG-IR), a non-insulin-based surrogate marker of insulin resistance, was calculated as Ln [(triglycerides, mg/dL × glucose, mg/dL)/2] [42].

A specific quantitative competitive ELISA immunoassay kit was used to measure plasma S1P levels in duplicate according to the manufacturer’s instructions (Cat# ELK8273, ELK Biotechnology Co., Ltd., Sugar Land, TX, USA). Eight patients’ samples were spiked with a fixed amount of the standard S1P and were assayed. In comparison to unspiked samples, the average recovery rate was 98.5% (range: 89–106%). The assay has a lower detection limit of 4.48 ng/mL, an inter-assay coefficient of variation (CV) < 10%, an intra-assay CV < 8%, and a recovery rate > 90%, and utilizes S1P pre-coated microplate, specific biotin-conjugated antibody, and Avidin conjugated to Horseradish Peroxidase (HRP) with its tetramethylbenzidine substrate and sulphuric acid to stop the reaction. Overnight fasting peripheral blood samples were aseptically collected in EDTA on ice to stabilize S1P [43]. The samples were centrifuged for 20 min at 4 °C and 1200× *g*, and the recovered plasma was stored in aliquots at −60 °C in the College’s Research lab until further use.

### 2.4. Statistical Analysis

IBM SPSS Statistics for Windows (version 27.0) and GraphPad Prism (version 9.0) were used for data analysis and visualization. Continuous variables were assessed for normality using the Shapiro–Wilk test and are presented as mean ± standard deviation (SD) for normally distributed data or median (range) for non-normally distributed data. Categorical variables are expressed as frequencies and percentages (n, %). For comparisons between two groups (T2DM patients versus healthy controls; patients with versus without complications), the independent samples *t*-test was used for normally distributed continuous variables, while the Mann–Whitney U test was applied for non-normally distributed variables. For comparisons across multiple groups (e.g., S1P quartiles, complication subtypes), the Kruskal–Wallis test with post hoc Dunn’s correction, or one-way ANOVA with Tukey’s post hoc test, was employed as appropriate. The chi-squared test (or Fisher’s exact test, where expected cell counts were <5) was used for categorical variables. Associations between plasma S1P levels and continuous variables were assessed using Pearson’s correlation coefficient (r) for normally distributed variables and Spearman’s rank correlation coefficient (ρ) for non-normally distributed variables.

Receiver Operating Characteristic (ROC) curve analysis was performed to evaluate the discriminatory ability of plasma S1P for distinguishing T2DM patients from healthy controls. The optimal cutoff value was determined using Youden’s index (sensitivity + specificity − 1), and the area under the curve (AUC) with 95% confidence interval (CI) was calculated. A multivariable binary logistic regression model was constructed to identify independent factors associated with the presence of diabetic complications. Variables with *p* < 0.10 in univariable analysis were entered into the model using forward stepwise selection. Results were reported as adjusted odds ratios (OR) with 95% CI.

To explore potential non-linear relationships between S1P and clinical parameters, patients were stratified into quartiles based on S1P levels (Q1: lowest; Q2: low-intermediate; Q3: high-intermediate; Q4: highest). Trends across the quartiles were assessed using the Jonckheere-Terpstra test for ordered alternatives. To formally test for U-shaped or J-shaped relationships, we employed two complementary approaches: (1) quadratic regression models including both linear (S1P) and quadratic (S1P^2^) terms, where a significant quadratic term indicated non-linearity; and (2) restricted cubic spline regression with three knots at the 10th, 50th, and 90th percentiles to statistically assess non-linear patterns. The complexity of these models was clinically justified to capture physiological homeostatic responses, which simple linear models often fail to detect. Model diagnostics, including residual analysis and goodness-of-fit assessments, were performed to ensure model validity. Moreover, utilizing both quadratic and spline approaches concurrently served as a robust methodological sensitivity analysis. Inflection points where the direction of association changed were identified from spline curves and quadratic model equations. Statistical significance was set at a two-tailed *p*-value of <0.05 for all analyses.

## 3. Results

The two groups of our study participants, healthy controls and patients, did not differ significantly in terms of sex distribution, age, and BMI. They differed significantly across all investigations, including S1P, HbA1c, and TyG insulin resistance index (*p* < 0.001), with the exception of WBCs, hemoglobin content, and LDL-C. The Mann–Whitney U test comparing males and females within each control and patient group revealed nonsignificant differences in plasma S1P levels. The Kruskal–Wallis test across age subgroups (<40, 40–60, and >60), within each control and patient group, revealed significantly higher S1P levels in the >60 subgroup of patients only (*p* = 0.047). Kruskal–Wallis tests across BMI subgroups (18.5–24.9, 25–29.9, and ≥30) within each control and patient group revealed nonsignificant differences in S1P levels (Table 1).

The distribution of the clinical characteristics and investigated findings among T2DM patients with and without complications is presented in Table 2. Parameters were markedly higher in those with complications, except for diastolic blood pressure, and had a borderline difference in BMI (*p* = 0.054). Patients with complications had a significantly higher systolic blood pressure (*p* = 0.008) and a higher prevalence of hypertension (*p* = 0.023). Sex and TC, LDL-C, Hb, RBCs, and WBCs did not show association with complications.

Plasma S1P levels in patients stratified by disease duration (<5, 5–10, and >10 years) showed significant progressive increases with longer disease duration (*p* = 0.021). Stratification for glycemic control (HbA1c, %; <7% and ≥7%) indicated that S1P levels were markedly higher in poorly controlled disease (*p* = 0.008). Stratifying patients by type, intensity, and insulin content (*p* < 0.001) treatment revealed progressive increases in plasma S1P from naïve, diet metformin, hypoglycemic to insulin, and combined treatment (*p* = 0.002) (Table 3). Insulin-treated patients had significantly higher S1P levels (1312.5 ng/mL) compared to those not on insulin (1218.5 ng/mL; *p* < 0.001). However, the chi-square test (or Fisher’s exact test where appropriate) analysis did not show a significant association between treatment modalities [(naïve, diet control, metformin, hypoglycemics, insulin, or combined (≥2 drugs)] and complication status; the nearest to significance was insulin therapy (*p* = 0.095). This likely reflects disease severity rather than a direct drug effect, as patients requiring insulin have more advanced disease. Oral hypoglycemic users also had non-significantly elevated S1P compared to untreated patients. Clear stepwise increase in S1P with increasing treatment intensity: No pharmacotherapy (1145.0 ng/mL), oral monotherapy (1228.8 ng/mL), and insulin-containing regimens (1308.7 ng/mL). This pattern strongly supports S1P as a marker for disease severity.

Table 4 presents variation in age, disease duration, HbA1c, systolic blood pressure, and S1P levels across the specific types of complications recorded, which reached significance only for S1P and systolic blood pressure, with a progressive trend from retinopathy, neuropathy, and nephropathy to mixed complications. Microvascular complications are presented, since we have only one case each of stroke and myocardial infarction among our patients, and hence could not be represented in the statistical analysis.

The trend of increases in plasma S1P with complications is further detailed in Table 5, where they were all significantly higher than in patients without complications, with an increasing trend from ophthalmopathy (*p* = 0.018), neuropathy (*p* = 0.007), nephropathy (*p* = 0.004), and mixed complications (*p* < 0.001). Mixed complications (≥2 types) had the highest S1P levels, which were significantly higher than those of single complications, establishing a dose–response relationship.

### 3.1. Spearman’s Correlation Analysis Among Investigations in Patients

Spearman’s correlation of S1P levels with clinical and laboratory investigations in patients and healthy controls, using Pearson’s correlation coefficient (r) and Spearman’s rank correlation coefficient (ρ), is presented in Table 6. Healthy controls did not show significant correlations. In patients, S1P was significantly positively correlated with age, disease duration, HbA1c, fasting blood glucose, TyG insulin resistance index, triglycerides, and blood pressure, but negatively correlated with HDL-C. Patients with complications have significantly higher systolic blood pressure and a higher prevalence of hypertension. S1P positively correlated with both systolic and diastolic blood pressure in T2DM patients, but not in controls. This finding suggests a potential link between S1P and hypertension in the diabetic state. S1P’s association with diabetes and its complications is independent of total cholesterol and hematological parameters (Hb, RBCs, and WBCs), which did not correlate significantly with S1P and did not differ between patients with and without complications, strengthening its potential as a specific biomarker.

### 3.2. Multivariable Binary Logistic Regression for Independent Factors Associated with T2DM Complications

Multivariable binary logistic regression for independent factors associated with T2DM complications presented in Table 7 indicated progressive stronger implications for HDL-C (*p* = 0.045), systolic blood pressure (*p* = 0.028), patients’ age (*p* = 0.018), insulin resistance (*p* = 0.017), glycemic control (*p* = 0.007), S1P (OR = 1.18 per 100 ng/mL increase, *p* = 0.003), and disease duration (*p* < 0.001).

### 3.3. Diagnostic Performance of S1P

The ability of plasma S1P variation to differentiate between T2DM patients and healthy controls as an independent risk factor was further confirmed by the area under the receiver operating characteristic curve (AUC/ROC), which showed significant but moderate diagnostic accuracy (*p* < 0.001 and AUC = 0.724). At an optimal plasma S1P cutoff value of 1142.5 ng/mL, a Youden’s index of 0.365, 95% confidence interval of 0.657–0.791, sensitivity was 71.4%, and specificity was 65.1% (Table 8).

### 3.4. Analysis of the Linear vs. Non-Linear U-/J-Shaped Quartile S1P Relationships

Given S1P’s dual roles in cytoprotection at physiological levels, vs. pro-inflammatory/pro-thrombotic effects at supra-physiological levels, analyzing the linear vs. non-linear (U-/J-shaped) relationship with S1P among the parameters investigated is biologically reasonable. After quartile (Q1 low, Q2 low-intermediate, Q3 high-intermediate, and Q4 high) stratification of patients, restricted cubic spline regression to assess non-linear patterns, threshold analysis to identify inflection points, where risk direction changes, and U-shaped linear vs. quadratic S1P^2^ model relationships were performed. Mixed complications show a J-shaped pattern, that is, complications are rare in low-to-intermediate S1P at low-to-intermediate S1P in Q1–Q2 but rise sharply above ~1250 ng/mL in Q4. In line with its negative correlation with HDL-C levels, S1P showed a non-linear, classic U-shaped pattern with HDL-C: highest HDL-C at intermediate S1P levels (~1150–1250 ng/mL) and decline at both low and high extremes. Mixed complications are rare at low-to-intermediate S1P levels but rise sharply above ~1250 ng/mL, forming a J-shaped pattern (Table 9).

Direct progressive linear relationships between S1P were confirmed with age, disease duration, systolic blood pressure, HbA1c, FBG, and TG, which increased with increases in S1P. A suggested optimal S1P range of 1100–1280 ng/mL appears to be the favorable metabolic and antidiabetic range, as it was associated with the highest HDL-C, lowest TyG insulin resistance index, and lowest complication risk, mixed complication rate, and composite metabolic risk (complications + hypertension + severe insulin resistance + low HDL). The relationships between HDL-C and composite metabolic risk are U-shaped, indicating that both low and high S1P are determinably associated with low HDL-C and unfavorable metabolic risk, with an optimal S1P range of 1120–1350 and 1100–1280 ng/mL, respectively. The relationships with each of TyG Index, complication risk, and mixed complication are J-shaped, with high S1P levels (above 1300, 1280, and 1250 ng/mL, respectively) exponentially associated with worse insulin resistance and microvascular complications. Progressive increases in S1P show a linear relationship with systolic blood pressure (Table 10).

## 4. Discussion

In this cross-sectional study, we investigated variations in plasma S1P levels in patients with T2DM compared to healthy controls and examined their associations with clinical characteristics, metabolic parameters, complications, and treatment modalities. Our principal findings are a follows: (1) plasma S1P is significantly elevated in T2DM patients; (2) S1P correlates positively with disease duration, glycemic control (HbA1c and FBG), insulin resistance (TyG index), triglycerides, and blood pressure, and negatively with HDL-C; (3) S1P levels increase progressively with complication burden, being highest in patients with nephropathy and mixed complications; (4) insulin-treated patients exhibit the highest S1P levels, reflecting disease severity; (5) S1P demonstrates moderate diagnostic accuracy for T2DM (AUC = 0.724); (6) multivariable analysis identifies S1P as an independent predictor of diabetic complications; and (7) novel non-linear U-shaped (with HDL-C, which substantiated the observed HDL-C negative correlation with S1P) and J-shaped (with complication risk) relationships reveal a suggested optimal S1P range of approximately 1100–1280 ng/mL, that requires validation in prospective studies.

Reportedly, obese humans and mice showed significantly higher plasma S1P levels, which positively correlated with body adiposity, total and LDL cholesterol, fasting insulin and insulin resistance, and HbA1c % [34,44]. The dyslipidemic free fatty acid overload characteristic of T2DM accelerates sphingolipid synthesis, shifting the ceramide/S1P rheostat toward S1P accumulation [3,4]. The positive correlations we observed between S1P and HbA1c, FBG, and TyG index suggest that hyperglycemia and insulin resistance promote S1P elevation, consistent with studies showing glucose-induced SphK activation in pancreatic β-cells and hepatocytes [16,17]. The inverse correlation and the U-shaped relationship with HDL-C are particularly noteworthy, as HDL is the primary carrier of apoM-bound S1P, and reduced HDL in T2DM may reflect impaired S1P binding capacity. Hence, it spares S1P for albumin binding [9,33,45,46].

A key finding of our study is the progressive increase in S1P with complication burden. Patients with complications had significantly higher S1P than those without, with a stepwise elevation from retinopathy to neuropathy to nephropathy, and the highest levels were in those with mixed complications. This “dose-dependent” pattern supports S1P as a cumulative marker of microvascular injury. Experimental studies have shown that S1P can exert both protective and detrimental effects in the kidney depending on receptor engagement. While apoM/S1P signaling via S1PR1/3 is reno-protective, albumin-bound S1P, activating S1PR2, promotes podocyte injury and fibrosis [27,28]. The observations that urinary S1P correlates with proteinuria [26] and that serum apoM inversely correlates with nephropathy stage [27] align with this duality. The elevated S1P in our nephropathy patients may reflect a shift toward albumin-bound S1P and S1PR2-mediated injury in advanced disease. In retinopathy, plasma S1P inversely correlates with disease severity in experimental models [29], and SphK inhibition protects against light-induced retinal degeneration [30]. S1P is an angiogenic factor through vascular endothelial growth factor (VEGF) and MMP-2 (matrix metalloproteinase-2) activation that implicates S1P in neovascular retinal disease [47]. Our observation of mild elevation in S1P in retinopathy patients suggests that circulating levels may not fully reflect local retinal S1P dynamics, or that compensatory elevations occur in response to vascular injury.

Our finding that S1P correlates positively with insulin resistance (TyG index) and negatively with HDL-C suggests that in T2DM, the balance may shift toward albumin-bound S1P as HDL-C declines and apoM content decreases [48]. This shift could explain why elevated total S1P in T2DM is associated with, rather than protective against, metabolic deterioration. The differential effects of S1P on carrier proteins—HDL/apoM versus albumin—provide a compelling explanation for the seemingly contradictory roles of S1P in diabetes [4,8]. HDL/apoM-bound S1P, signaling through S1PR1/S1PR3, promotes endothelial barrier function, eNOS activation, and insulin sensitivity. In contrast, albumin-bound S1P preferentially activates S1PR2, inducing pro-inflammatory and insulin-antagonistic responses [34,35].

The positive correlation between S1P and blood pressure, particularly systolic blood pressure, is another important observation. S1P regulates vascular tone through S1PR2- and S1PR3-mediated effects on endothelial function and smooth muscle contraction [8]. In diabetes, where endothelial dysfunction is prevalent, elevated S1P may contribute to increased vasoconstriction and hypertension. The higher prevalence of hypertension in patients with complications further supports a link between S1P, vascular dysfunction, and microvascular injury. The stepwise increase in S1P from untreated patients (1145.0 ng/mL) through oral monotherapy (1228.8 ng/mL) to insulin-containing regimens (1308.7 ng/mL) likely reflects disease severity rather than direct drug effects. Patients requiring insulin have longer disease duration, poorer glycemic control, and higher complication rates—all associated with elevated S1P. While some antidiabetic drugs may modulate sphingolipid metabolism [5,49,50], our data suggest that S1P elevation is primarily a marker of advanced disease rather than a treatment-specific effect, as the relationships had marginal significance.

Perhaps our most novel finding is the demonstration of non-linear relationships between S1P and key metabolic parameters. The U-shaped relationship with HDL-C—with the highest HDL-C at intermediate S1P (1100–1350 ng/mL) and lower HDL-C at both extremes—suggests that both insufficient and excessive S1P may be detrimental to lipoprotein metabolism or vice versa. The J-shaped relationship with complication risk, with a sharp increase above ~1250 ng/mL, indicates a threshold effect where S1P transitions from compensatory to pathogenic. These patterns align with emerging evidence from cardiovascular populations, where U-shaped associations between each of plasma HDL-C and S1P vs. risk for cardiovascular disease and mortality have been reported [40,51], and from S1P lyase deficiency, where marked S1P elevation causes multi-organ dysfunction [41]. The identification of an optimal S1P window (1100–1280 ng/mL) associated with the most favorable metabolic profile—highest HDL-C, lowest insulin resistance, and lowest complication rates—has important therapeutic implications. It suggests that S1P-modulating therapies should aim to maintain levels within this range rather than simply increasing or decreasing S1P indiscriminately. Previous publications reported conflicting results. Plasma S1P levels are elevated in T2DM [23,52]. The observed reductions in plasma levels of S1P in T2DM, compared to those in healthy controls, were related to the extent of glycemic and lipemic control and did not differ between those with normal albuminuria or macro-albuminuria [53]. Reductions in S1P content of plasma HDL inversely correlate with HbA1c in T2DM [32,45].

ROC analysis demonstrated that plasma S1P has moderate diagnostic accuracy for T2DM (AUC = 0.724), with sensitivity (71.4%) and specificity (65.1%) comparable to other emerging biomarkers. More importantly, the independent association of S1P with complications in multivariable regression (OR = 1.18 per 100 ng/mL, *p* = 0.003) suggests prognostic value beyond traditional risk factors. The inclusion of S1P alongside established predictors (age, disease duration, HbA1c, TyG index, HDL-C, systolic blood pressure) in the regression model strengthens its potential as a complementary biomarker for risk stratification. Early postpartum reduction in plasma ceramide biosynthesis, implicating ceramide synthase 2 and 4, contributes to pancreatic β-cell dysfunction and transition of Hispanic women with pregnancy diabetes to T2DM [50]. The resultant accumulation of S1P levels could be the culprit effector. Oppositely, serum S1P level was reported to be highest in healthy controls, decreased in pre-diabetes, and further decreased upon T2DM development, and predicted an elevated risk of cardiovascular complications in patients. S1P levels were associated with sex and lean mass index, but not with BMI [54]. Compared with lean healthy subjects, obese subjects had significantly elevated plasma S1P levels that were positively correlated with the percentage of BMI, waist circumference, total body fat, insulin resistance, fasting insulin, HbA1c, and total and LDL-C but not HDL-C [5,34]. Adiponectin receptor has inherent ceramidase activity that, upon activation, increases tissue sphingosine and S1P content. This does not happen in T2DM due to the low levels of adiponectin [49].

Strengths of this study include the well-characterized cohort, comprehensive clinical and biochemical phenotyping, inclusion of a matched healthy control group, detailed analysis of complication subtypes, and systematic evaluation of non-linear relationships. The sample size was adequately powered, and rigorous statistical methods were employed. Limitations warrant consideration. First, the cross-sectional design, as a snapshot, precludes causal inferences, the assessment of temporal relationships, and the inference of directionality. And, the findings may not be easily generalized into a global perspective. Second, we measured total plasma S1P rather than carrier-specific fractions (HDL/apoM-bound vs. albumin-bound), which may have different biological implications. Third, the potential confounding effects of diet variations, comorbid therapies such as statins and antihypertensives, and physical activity were not accounted for. Fourth, the relatively small number of patients in some complication subgroups (particularly nephropathy only, n = 8) limits statistical power for subgroup analyses. Fifth, we did not measure sphingosine kinase activities, S1P receptor expression, catabolic enzymes, or downstream signaling molecules that could provide mechanistic insights. Sixth, the single-center design may limit generalizability to other populations. Seventh, although the population had a predominantly sedentary lifestyle, high-energy and moderate-to-low salt consumption, the confounding effects of diet (particularly salt content), antihypertensive treatments, physical activity, and statins were not adjusted for, which potentially led to residual confounding.

## 5. Conclusions

Plasma S1P is significantly elevated in T2DM patients compared to healthy controls and correlates with key indicators of disease severity, including duration, treatment intensity, poor glycemic control, insulin resistance, dyslipidemia, hypertension, and the presence and extent of microvascular complications. The progressive increase in S1P from patients without complications through those with retinopathy, neuropathy, and nephropathy to those with mixed complications supports its role as a cumulative marker of diabetic tissue injury. We identified non-linear U-shaped and J-shaped relationships between S1P, HDL-C, and diabetic microvascular complications that revealed an optimal S1P window of approximately 1100–1280 ng/mL associated with the most favorable metabolic profile. S1P demonstrated moderate diagnostic accuracy for T2DM (AUC = 0.724) and emerged as an independent predictor of diabetic complications in multivariable analysis (OR = 1.18 per 100 ng/mL), suggesting prognostic value beyond traditional risk factors. Nephropathy is associated with the highest S1P levels among specific complications, followed by neuropathy and retinopathy. Mixed complications show a “dose-dependent” increase in S1P, supporting its role as a cumulative severity marker. Future longitudinal studies are warranted to establish the predictive value of S1P for incident complications and to explore the mechanistic basis of the observed non-linear relationships.

## Figures and Tables

**Table 1:** Demographic and clinical characteristics of study participants: type 2 diabetic (T2DM) and healthy controls.

Variable	Controls (n = 63)	Patients (n = 140)	*p* Value
Gender (Male)	28 (44.4%)	52 (37.1%)	0.327 *
Age (Years)	52 (24–80)	55 (23–82)	0.127
BMI (kg/m^2^)	29.8 (20.7–54.4)	31.2 (20.0–58.6)	0.121
HbA1c (%)	5.4 (4.8–8.1)	7.9 (5.0–15.1)	**<0.001**
FBG (mM/L)	5.0 (3.8–6.8)	7.6 (4.1–22.2)	**<0.001**
TyG Index	8.31 ± 0.36	9.24 ± 0.68	**<0.001 ^#^**
TGs (mM/L)	1.08 (0.50–3.21)	1.67 (0.54–8.59)	**<0.001**
TC (mM/L)	5.20 (2.21–7.53)	4.58 (1.10–8.15)	**0.004**
HDL-C (mM/L)	1.45 (0.51–2.21)	1.24 (0.70–2.42)	**<0.001**
LDL-C (mM/L)	2.96 (1.35–5.73)	2.71 (0.64–6.85)	0.085
Hb (g/dL)	13.8 (7.4–18.0)	13.9 (4.5–19.4)	0.674
RBCs (×10^6^/μL)	4.81 ± 0.52	5.03 ± 0.64	**0.038 ^#^**
WBCs (×10^3^/μL)	6.84 (3.9–13.9)	7.58 (3.9–13.4)	0.092
S1P (ng/mL)	1075.1 (202.0–1510.0)	1256.7 (149.4–1510.0)	**<0.001**

Data shown are frequency n (%), median (range) or mean ± SD, and *p*-value for chi-square *, *t*-test ^#^, or Mann–Whitney U. BMI = body mass index; HbA1c = hemoglobin A1c; TyG = triglycerides–glucose insulin resistance index; TGs = triglycerides; TC = total cholesterol; HDL-C = HDL cholesterol; LDL-C = LDL cholesterol; Hb = hemoglobin; RBCs = erythrocyte count; WBCs = leukocyte count; and S1P = sphingosine-1-phosphate.

**Table 2:** Characteristics of T2DM patients stratified by complication status.

Variable	Complications		*p* Value
	No (n = 92)	Yes (n = 48)	
Age (years)	53 (23–80)	59 (31–82)	**0.003**
BMI (kg/m^2^)	30.8 (20.0–58.6)	32.4 (22.3–55.3)	0.054
Disease Duration (years)	8 (0.1–30)	15 (0.8–32)	**<0.001**
HbA1c (%)	7.6 (5.0–13.9)	8.5 (5.7–15.1)	**0.002**
FBG (mM/L)	7.2 (4.1–20.5)	8.9 (4.3–22.2)	**0.007**
TyG Index	9.12 ± 0.62	9.51 ± 0.71	**0.007** *
TGs (mM/L)	1.58 (0.54–4.49)	1.92 (0.70–8.59)	**0.012**
HDL-C (mM/L)	1.28 (0.82–2.42)	1.16 (0.70–2.21)	**0.028**
Systolic BP (mmHg)	130 (94–179)	138 (108–179)	**0.008**
Diastolic BP (mmHg)	80 (56–100)	82 (58–99)	0.124
Hypertension (BP ≥ 140/90)	28 (30.4%)	24 (50.0%)	**0.023 ^#^**
S1P (ng/mL)	1205.7 (149.4–1510.0)	1312.5 (439.4–1510.0)	**0.001**

Data shown are median (range), mean ± SD or frequency n (%), and *p*-value for Mann–Whitney U, *t*-test * or chi-square ^#^. BP = blood pressure. Other abbreviations are as defined in Table 1.

**Table 3:** Plasma sphingosine-1-phosphate (S1P) levels in T2DM patients stratified for (1) disease duration, (2) glycemic control (HbA1c, %), (3) type of treatment, (4) treatment intensity, and (5) insulin vs. non-insulin treatment.

Variable		n	S1P (ng/mL)	*p* Value
Disease duration	<5 years	38	1199.4 (452.1–1510.0)	**0.021** *
	5–10 years	42	1248.7 (586.0–1510.0)	
	>10 years	60	1285.4 (149.4–1510.0)	
Glycemic control (HbA1c %)	<7%	42	1183.4 (452.1–1510.0)	**0.008**
	≥7%	98	1279.0 (149.4–1510.0)	
Type of treatment	Naïve (no treatment)	10	1129.2 (586.0–1253.5)	Reference
	Diet control only	7	1159.5 (1071.9–1358.7)	0.412
	Metformin only	52	1212.0 (452.1–1462.2)	**0.038**
	Oral hypoglycemics only	16	1248.7 (1016.1–1449.5)	**0.021**
	Insulin only	24	1312.5 (1075.1–1510.0)	**<0.001**
	Combination therapy (≥2 drugs)	31	1299.7 (1126.0–1510.0)	**0.002**
Treatment intensity	No pharmacotherapy (naïve/diet)	17	1145.0 (586.0–1358.7)	Reference
	Oral monotherapy	68	1228.8 (452.1–1462.2)	**0.027**
	Insulin-containing regimens	55	1308.7 (1075.1–1510.0)	**<0.001**
Insulin treatment	No	85	1218.5 (452.1–1499.0)	**<0.001**
	Yes	55	1308.7 (1075.1–1510.0)	

Data shown are median (range), and *p*-value for Kruskal–Wallis * or Mann–Whitney U. Abbreviations are as defined in Table 1.

**Table 4:** Clinical characteristics of T2DM patients stratified by specific complication types.

Variable	Ophthalmopathy (n = 12)	Neuropathy (n = 14)	Nephropathy (n = 8)	Mixed (n = 14)	*p* Value
Age (Years)	58 (45–71)	57 (43–68)	61 (52–75)	62 (47–82)	0.182
Disease Duration (Years)	14 (6–25)	15 (7–26)	18 (10–30)	20 (10–32)	0.064
HbA1c (%)	8.7 (6.9–12.0)	8.9 (7.0–13.9)	9.2 (7.3–12.6)	9.8 (7.5–15.1)	0.098
Systolic BP (mmHg)	136 (118–160)	134 (116–158)	148 (130–179)	152 (128–179)	**0.015**
S1P (ng/mL)	1291.3 (1126.0–1462.2)	1308.7 (1167.5–1478.2)	1331.6 (1236.0–1449.5)	1355.5 (1248.7–1510.0)	**0.032**

Data shown are median (range), and *p*-value for the Kruskal–Wallis test. S1P = sphingosine-1-phosphate. Abbreviations are as defined in Table 1.

**Table 5:** Comparison of sphingosine-1-phosphate (S1P) plasma levels among the specific T2DM complication types.

Complication Type	n	S1P (ng/mL) Median (Range)	vs. Oph	vs. Neph	vs. Neur	vs. No Comp
No Complications (Comp)	92	1205.7 (149.4–1510.0)	**0.018**	**0.004**	**0.007**	Reference
Ophthalmopathy (Oph) Only	12	1291.3 (1126.0–1462.2)	—	0.412	0.528	**0.018**
Nephropathy (Neph) Only	8	1331.6 (1236.0–1449.5)	0.412	—	0.673	**0.004**
Neuropathy (Neur) Only	14	1308.7 (1167.5–1478.2)	0.528	0.673	—	**0.007**
Mixed Comp (≥2 types)	14	1355.5 (1248.7–1510.0)	**0.048**	0.215	0.094	**<0.001**

Data shown are median (range) and *p*-value for the Mann–Whitney U test.

**Table 6:** Correlation of sphingosine-1-phosphate with clinical and laboratory investigations among T2DM patients and healthy controls.

Variable	Healthy Controls		T2DM Patients	
	r/ρ	*p* Value	r/ρ	*p* Value
Age	0.124 ^1^	0.331	0.218 ^1^	**0.010**
BMI	−0.087 ^1^	0.498	0.156 ^1^	0.065
Disease Duration	—	—	0.297 ^1^	**<0.001**
HbA1c	0.152 ^1^	0.233	0.342 ^1^	**<0.001**
FBG	0.108 ^1^	0.399	0.297 ^1^	**<0.001**
TyG Index	0.176 ^2^	0.168	0.384 ^2^	**<0.001**
TGs	0.139 ^1^	0.277	0.356 ^1^	**<0.001**
TC	−0.065 ^1^	0.611	−0.089 ^1^	0.298
HDL-C	−0.143 ^1^	0.263	−0.211 ^1^	**0.012**
LDL-C	−0.052 ^1^	0.684	−0.064 ^1^	0.452
Hb	0.088 ^1^	0.492	−0.042 ^1^	0.623
RBCs	0.112 ^2^	0.381	0.078 ^2^	0.359
WBCs	0.157 ^1^	0.218	0.124 ^1^	0.145
Systolic BP (mmHg)	0.087 ^1^	0.498	0.234 ^1^	**0.006**
Diastolic BP (mmHg)	0.052 ^1^	0.684	0.189 ^1^	**0.026**
Pulse Pressure (SBP-DBP)	0.063 ^1^	0.621	0.201 ^1^	**0.018**

Data shown are Spearman’s rank correlation coefficient (ρ) ^1^, Pearson’s correlation coefficient (r) ^2^, and their *p*-values.

**Table 7:** Multivariable binary logistic regression for independent factors associated with T2DM complications.

Variable	Adjusted Odds Ratios (OR)	95% Confidence Interval (CI)	*p* Value
HDL-C (mM/L)	0.42	0.18–0.98	**0.045**
Systolic Blood Pressure (per 10 mmHg)	1.21	1.02–1.44	**0.028**
Age (per year)	1.04	1.01–1.08	**0.018**
TyG Index	1.89	1.12–3.19	**0.017**
HbA1c (%)	1.32	1.08–1.61	**0.007**
S1P (per 100 ng/mL)	1.18	1.06–1.32	**0.003**
Disease Duration (per Year)	1.09	1.04–1.15	**<0.001**

Notes: Model adjusted for age, sex, BMI, disease duration, HbA1c, TyG index, TG, HDL-C, systolic blood pressure, and sphingosine-1-phosphate (S1P).

**Table 8:** Receiver operating characteristic (ROC) curve diagnostic performance of the variation in sphingosine-1-phosphate levels for T2DM.

Parameter	Value
Area Under Curve (AUC)	0.724
95% Confidence Interval	0.657–0.791
*p*-Value	**<0.001**
Optimal Cutoff Value	1142.5 ng/mL
Sensitivity	71.4%
Specificity	65.1%
Youden’s Index	0.365

**Table 9:** Clinical characteristics and clinical outcomes across sphingosine-1-phosphate quartiles (n = 35 each) in T2DM patients.

Variable	Q1 (Lowest)	Q2 (Low-Intermediate)	Q3 (High-Intermediate)	Q4 (Highest)	*p*-Value	Pattern
	998.6 (149.4–1125.3)	1193.0 (1126.0–1248.7)	1291.3 (1248.8–1338.0)	1424.0 (1338.1–1510.0)		
Age (Years)	51 (23–78)	55 (31–80)	58 (35–82)	62 (39–82)	**0.008**	Linear ↑
BMI (kg/m^2^)	31.2 (22.3–48.0)	30.8 (23.6–44.7)	31.5 (24.1–58.6)	32.8 (25.4–55.3)	0.124	Plateau
Disease Duration (Years)	8 (0.1–25)	10 (0.5–26)	12 (1–30)	15 (2–32)	**0.003**	Linear ↑
HbA1c (%)	7.4 (5.0–11.5)	7.8 (5.5–12.6)	8.3 (5.8–13.9)	9.1 (6.2–15.1)	**0.001**	Linear ↑
FBG (mM/L)	6.8 (4.1–14.6)	7.3 (4.3–15.5)	8.1 (4.9–19.7)	9.5 (5.2–22.2)	**0.002**	Linear ↑
TyG Index	8.94 ± 0.58	9.16 ± 0.63	9.38 ± 0.66	9.67 ± 0.71	**<0.001 ^#^**	Linear ↑
TG (mM/L)	1.28 (0.54–3.12)	1.56 (0.62–4.49)	1.83 (0.70–5.76)	2.31 (0.89–8.59)	**<0.001**	Linear ↑
TC (mM/L)	4.51 (1.10–6.89)	4.62 (2.21–7.18)	4.58 (2.43–7.53)	4.49 (2.07–8.15)	0.876	Flat
HDL-C (mM/L)	1.38 (0.89–2.42)	1.29 (0.82–2.21)	1.18 (0.72–1.98)	1.09 (0.70–1.83)	**0.005**	Linear ↓
LDL-C (mM/L)	2.63 (0.64–4.87)	2.71 (1.13–5.21)	2.74 (1.35–5.73)	2.68 (1.02–6.85)	0.892	Flat
Systolic BP (mmHg)	128 (94–160)	132 (108–169)	138 (115–179)	148 (116–179)	**0.001**	Linear ↑
Diastolic BP (mmHg)	78 (56–95)	80 (62–98)	82 (64–99)	86 (58–100)	0.058	Trend ↑
No Complications	29 (82.9%)	26 (74.3%)	21 (60.0%)	16 (45.7%)	**0.003** *	Linear ↓
Any Complication	6 (17.1%)	9 (25.7%)	14 (40.0%)	19 (54.3%)	**0.003** *	Linear ↑
Ophthalmopathy	2 (5.7%)	3 (8.6%)	4 (11.4%)	3 (8.6%)	0.842 *	Flat
Nephropathy	1 (2.9%)	1 (2.9%)	2 (5.7%)	4 (11.4%)	0.312 *	Trend ↑
Neuropathy	2 (5.7%)	3 (8.6%)	4 (11.4%)	5 (14.3%)	0.618 *	Trend ↑
Mixed (≥2 types)	1 (2.9%)	2 (5.7%)	4 (11.4%)	7 (20.0%)	**0.041** *	J-shaped

Data shown are median (range), mean ± SD or frequency n (%), and *p*-value for chi-square test *, ANOVA ^#^ or Kruskal–Wallis for trend across quartiles. Trends across quartiles were assessed using the Jonckheere–Terpstra test for ordered alternatives.

**Table 10:** Test for U-shaped linear vs. quadratic (sphingosine-1-phosphate)^2^ model relationships in T2DM patients.

Variable	Linear Model (S1P) *p*-Value	Quadratic Model (S1P^2^) *p*-Value	Best Fit Shape	Inflection Point (ng/mL)
Age	**0.010**	0.342	Linear	—
BMI	0.065	0.078	Flat/Weak U	~1120
Disease Duration	**<0.001**	0.412	Linear	—
HbA1c	**<0.001**	0.208	J-shaped	~1150
TyG Index	**<0.001**	0.094	J-shaped	~1150
Triglycerides	**<0.001**	0.112	Linear	—
HDL-C	**0.012**	0.087	U-shaped	~1100, ~1350
Systolic Blood Pressure	**0.006**	0.124	Linear	—
Complication Risk	**<0.001**	**0.032**	U-shaped	~1120, ~1300
Mixed Complications	**<0.001**	**0.018**	J-shaped	~1250

Abbreviations are as defined in Table 1.

## Data Availability

All of the biostatistically analyzed data are presented in the manuscript. The spreadsheet of the raw data is available upon reasonable request, subject to proper local bioethical approval.

## Abbreviations

apoM	apolipoprotein M
AUC	area under the ROC curve
BMI	body mass index
BP	blood pressure
eNOS	endothelial nitric oxide synthase
FBG	fasting blood glucose
Hb	hemoglobin
HbA1c	hemoglobin A1c
HDL-C	high-density lipoprotein cholesterol
IL	interleukin
LDL-C	low-density lipoprotein cholesterol
MMP-2	matrix metalloproteinase-2
OR	odds ratio
RBCs	erythrocyte count
ROC	receiver operating characteristic curve
S1P	sphingosine-1-phosphate
S1PR	S1P receptor
SphK	sphingosine kinase
T2DM	type 2 diabetes mellitus
T1DM	type 1 diabetes mellitus
TC	total cholesterol
TyG	triglycerides–glucose
VEGF	vascular endothelial growth factor
WBCs	leukocyte count
